# Expression patterns of signalling molecules and transcription factors in the early rabbit embryo and their significance for modelling amniote axis formation

**DOI:** 10.1007/s00427-021-00677-w

**Published:** 2021-06-07

**Authors:** Ruben Plöger, Christoph Viebahn

**Affiliations:** Institute of Anatomy and Embryology, University Medical Center Göttingen, Göttingen, Germany

**Keywords:** Germ layers, Primitive streak, Twinning, T-box transcription factors, Wnt-signalling, Rabbit

## Abstract

The anterior-posterior axis is a central element of the body plan and, during amniote gastrulation, forms through several transient domains with specific morphogenetic activities. In the chick, experimentally proven activity of signalling molecules and transcription factors lead to the concept of a ‘global positioning system’ for initial axis formation whereas in the (mammotypical) rabbit embryo, a series of morphological or molecular domains are part of a putative ‘three-anchor-point model’. Because circular expression patterns of genes involved in axis formation exist in both amniote groups prior to, and during, gastrulation and may thus be suited to reconcile these models, the expression patterns of selected genes known in the chick, namely the ones coding for the transcription factors eomes and tbx6, the signalling molecule wnt3 and the wnt inhibitor pkdcc, were analysed in the rabbit embryonic disc using in situ hybridisation and placing emphasis on their germ layer location. Peripheral *wnt3* and *eomes* expression in all layers is found initially to be complementary to central *pkdcc* expression in the hypoblast during early axis formation. *Pkdcc* then appears — together with a posterior-anterior gradient in *wnt3* and *eomes* domains — in the epiblast posteriorly before the emerging primitive streak is marked by *pkdcc* and *tbx6* at its anterior and posterior extremities, respectively. Conserved circular expression patterns deduced from some of this data may point to shared mechanisms in amniote axis formation while the reshaping of localised gene expression patterns is discussed as part of the ‘three-anchor-point model’ for establishing the mammalian body plan.

**Supplementary Information:**

The online version contains supplementary material available at 10.1007/s00427-021-00677-w.

## Introduction

As forerunner of the cranio-caudal body axis, the anterior-posterior (a.-p.) axis of the vertebrate embryo emerges through a series of transient morphological hallmarks and molecularly defined zones during early gastrulation (see Wolpert et al. 2015). Hallmarks and zones are considered spatiotemporally unique when a single individual develops from one egg, and some of them may duplicate when identical twins develop, either physiologically as in the armadillo (Newman and Patterson 1910) or experimentally as in the sea urchin (Driesch 1892) or the chick (Lutz 1949; Torlopp et al. 2014). During evolution, amniotic embryos have retained several axial hallmarks, one prominent example being the primitive streak (PS), the morphological indicator of the posterior pole and of mesoderm formation (Bellairs 1986). Other axial hallmarks, however, appear to be class-specific: In mammals, the first morphological sign of the a.-p. axis is found at the anterior pole at a late pre-gastrulation stage and may be called, for example, anterior visceral endoderm (AVE) in the mouse, anterior marginal crescent (AMC) in the rabbit (see Viebahn 1999) or anterior visceral hypoblast (AVH) in cattle (van Leeuwen et al. 2015); whatever their name, the appearance of these hallmarks is followed by an elongation of the embryonic disc at the posterior pole to create the territory for the primitive streak. In the avian model organism, the chick (see Stern 2004), in contrast, the first morphological sign of the a.-p. axis appears at the posterior pole, is best known as Koller’s sickle (Koller 1882), and carries in itself both the region and the progenitors of the PS (see Izpisua-Belmonte et al. 1993; Bachvarova et al. 1998). As both mammals and birds have principally a flat embryonic disc at this stage of development, early mammalian a.-p. axis formation can be addressed as being inverted in comparison to the avian embryo, but, intriguingly, several genes involved are evolutionarily conserved between these two vertebrate classes, and some of them exhibit inverted expression patterns congruent with these morphological distinctions (see Plöger and Viebahn 2018). Furthermore, morphogenetic movements in the early mammalian embryonic disc show oriented cell divisions and ‘processional’ cell movements bringing cells of the former posterior region preferentially into the centre of the elongating disc (Viebahn et al. 2002; Stankova et al. 2015), whereas in the chick, most cells of the former posterior region remain posteriorly due to a specific polonaise-like cell movement (Gräper 1929; Wetzel 1929; for review, see Serrano Nájera and Weijer 2020).

Differences in morphology, gene expression patterns and cell movements lead to dedicated testable models which may explain the development of the a.-p. polarity, bilateral symmetry and, possibly, twinning in those vertebrate species where maternal determinants are unlikely to play a determining role (see Arias et al. 2017). One of these models is the global positioning system (GPS) for birds (Bertocchini and Stern 2012), where the anterior and posterior poles of the pre-gastrulation embryonic disc are defined, possibly following a paracrine growth factor influence (Arias et al. 2017), in a comparatively large, single-layer and homogenous array of cells. Specifically, the GPS is characterised by the independent regulation of *gata2* and *vg1* transcription at the anterior and posterior poles, respectively: *Gata2* (coding for a transcription factor) appears to be localised slightly early than *vg1* (coding for a TGFß growth factor) and introduces an anterior bias rather than suppressing primitive streak formation; localised *vg1*, in contrast, is directly necessary and sufficient to form the primitive streak (Bertocchini and Stern 2012). Overall, axis formation of the chick consists of genes which either mark the posterior pole and have an activating function in PS formation (Seleiro et al. 1996; Chapman et al. 2002; Torlopp et al. 2014; Lee et al. 2020) or which mark the anterior pole and have an inhibitory function in PS formation (Arias et al. 2017; Torlopp et al. 2014). In contrast, circular expression patterns in the peripheral embryonic disc marking the entire marginal zone are seen for genes such as *wnt8c*, coding for a signalling molecule, or *tbx6*, coding for a T-box transcription factor (Lee et al. 2020); mathematical modelling of breaking radial symmetry by a uniform-to-polarised transformation of *bmp4* and *vg1* expression (Arias et al. 2017) indicates the existence of a concentric gene expression system prior to global positioning in which two groups of axially active moieties seem to be involved. The interaction between activating genes of the ‘dual’ GPS and genes of the concentric system normally initiates PS formation in the posterior marginal zone (pMZ) (cf. Skromne and Stern 2001) or, indeed, formation of multiple PSs (and thus twinning) in the case of misexpression of activating genes in ectopic parts of the marginal zone (Shah et al. 1997; Skromne and Stern 2001). In support of this sequence of events, the ring-like patterns of *wnt8c* and *tbx6* (Torlopp et al. 2014; Lee et al. 2020), on the one hand, develop a marked posterior-to-anterior gradient originating from the posterior pole, while *eomes*, a conserved T-box-related transcription factor heavily involved in early axis related events (Arnold et al. 2008; van Leeuwen et al. 2015), on the other hand, shows a peripheral, partially extraembryonic, ring-like pattern in addition to its posterior expression domain in the pMZ (Pernaute et al. 2010).

For mammals, a tentative three-anchor-point (TAP) model was developed in the rabbit on the basis of the mammotypic morphology and a couple of intriguing gene expression patterns (Plöger and Viebahn 2018). In contrast to the GPS, the TAP model starts from a small, with epiblast and hypoblast already bilayered, pre-gastrulation array of cells (cf. Stern and Downs 2012) and introduces the need for three anchor points to stabilise the position of the a.-p. axis during a phase of vigorous growth of the embryo; the three anchor points are successively dotted along a straight line running through the future anterior and posterior poles and ‘gradually’ polarise the embryonic disc with the effect that the PS, the irreversible sign of the a.-p. axis, is held in position by the two last-appearing anchor points. The first of these anchor points is the AMC, with its high cell density and cuboidal hypoblast in a wide part of the margin at the future anterior pole at stage 1 (Viebahn et al. 1995a). At the posterior pole, the embryonic disc elongates at stage 2 through the formation of the posterior gastrula extension (PGE, Viebahn et al. 2002). The PGE has a characteristic cuboidal epiblast epithelium as compared to the higher epiblast epithelium of the remaining embryonic disc which is now addressed as the anterior gastrula plate (AGP, Plöger and Viebahn 2018). The morphological border between PGE and AGP is straddled by a sickle-shaped expression domain of *nodal* (Yoshida et al. 2016; Plöger and Viebahn 2018) which codes for an evolutionarily conserved growth factor central to axis formation (Conlon et al. 1991; Varlet et al. 1997; Bertocchini and Stern 2002; Perea-Gomez et al. 2002). During the transition to stage 3, this wide *nodal* domain condenses and coincides with the future region of the anterior part of the PS and was thus named anterior streak domain (ASD; Plöger and Viebahn 2018). As the ASD condenses, it is considered to act as a second anchor by gradually fixing the anterior extremity of the rising PS to the midline in the PGE. The third and most posterior anchor point arises in the posterior part of the PGE, which elongates fully during stage 3, and is defined by bottle cells and first mesoderm cells (Viebahn et al. 1995b). This successive development of three anchor points thus contrasts with the ‘dual’ GPS in the avian GPS in allowing a tight control of axis formation rather than introducing only an a.-p. bias.

Interestingly, mammals may share some similarities with birds in the case of concentric molecular control system: simple ring-like expression patterns are known of *bmp2* and *bmp4* in context of germline development in the mouse (bmp4: Lawson et al. 1999; bmp2: Coucouvanis and Martin 1999; Ying and Zhao 2001) and rabbit (Hopf et al. 2011) or of *pitx2* in the context of the early axis formation in the rabbit (Plöger and Viebahn 2018). In the mouse, in addition, extra- and intraembryonic expression of *wnt3* (Liu et al. 1999; Rivera-Perez and Magnuson 2005), *eomes* (Ciruna and Rossant 1999; Russ et al. 2000; Nowotschin et al. 2013) and possibly *brachyury* (Thomas and Beddington 1996) show a ring-like pattern which is best seen when the egg cylinder shape typical of rodents is schematically flattened into a disc (Behringer et al. 2000); amongst the genes with a circular expression pattern, however, *tbx6* seems to show a clear difference between amniote classes as its expression seems to be absent prior to its expression in the murine PS (Chapman et al. 1996), while *wnt3* seems to start with a sickle-shaped pattern at mid-axis formation in the rabbit (Yoshida et al. 2016).

The interrelationship between the GPS, the TAP model and a putative generalised concentric molecular system of amniote axis formation suggests that examining some key molecular players, not least the ones found in the large screen for axis related genes performed in the chick (Torlopp et al. 2014), may be suited to find molecular mechanisms underlying the TAP model, the least established of these model systems. For testing the relationship between the TAP model and a putative concentric system, *wnt3*, *tbx6* and *eomes* seem to be interesting because of their ring-like expression patterns in chick or mouse during early axis formation. The *pkdcc* gene, in contrast, seems interesting because of its expression in the mouse in a location possibly homologous to the ASD (Imuta et al. 2009) and because of its function as a negative regulator of the wnt pathway (Vitorino et al. 2015; Ding et al. 2017). These four genes are, therefore, analysed here using in situ hybridisation and histological sections in the rabbit embryo. The results reveal *eomes* and, indeed, *wnt3* to belong to the group of genes showing a ring-like expression pattern as a sign of evolutionarily conserved axis formation in amniotes; *tbx6* shows a ‘dual’ pattern marking first the AMC as the first (anterior) anchor point and later the (posterior) third anchor point; *pkdcc* and *wnt3*, finally, show almost complementary expression patterns as a sign of a possible functional connection between the concentric system and the three anchor point model, thus reconciling different models of axis formation in the amniote embryo.

## Methods

### Animals and tissue

In accordance with the ethical standards of the German ‘Tierschutzgesetz’, embryos were collected from young adult New Zealand White rabbits (Charles River, Germany) following a well-established protocol (Püschel and Jouneau 2014): At 5.0–6.5 days after natural mating, i.e. 5.0–6.5 days post-coitum (dpc), the embryos reached the developmental stages needed for this study, so that a lethal dose (1.25–2.5 ml/kg body weight) of Narcoren ® (Merial, Lyon, France) was injected intravenously in the maternal rabbit. After the exposure of the uteri, the uterine horns were flushed using warm phosphate-buffered saline (PBS) to collect the embryos, called blastocysts at this stage. These blastocysts were washed, fixed for 1 h in 4% paraformaldehyde (PFA) in phosphate buffer, freed from the zona pellucida and then dissected so that the embryonic disc surrounded by extraembryonic tissue could be flattened to define the stage on the basis of morphological criteria (see below). The dissected embryos were dehydrated and stored in ethanol at −20 °C unless they were directly used for whole-mount in situ hybridisation.

### Staging

Morphological criteria of the early rabbit embryo were used for staging as follows. Stage 0 covers the long period before the first morphological sign of the anterior-posterior axis appears and is defined by an irregular border and a uniform cell density of the embryonic disc which consists of three cell layers, i.e., the polar trophoblast (also known as Rauber’s layer [Rauber 1875]), the epiblast, and the hypoblast. The polar trophoblast and the hypoblast continue as extraembryonic mural trophoblast and yolk sac epithelium, respectively, towards the abembryonic pole of the blastocyst. At stage 1, the embryonic disc, now almost oval shaped, shows a distinct margin and higher cell density anteriorly than posteriorly, which, combined with the histological proof of cuboidal hypoblast cells, all define the first sign of the anterior-posterior axis, the anterior marginal crescent (AMC, Viebahn et al. 1995a). At stage 2, the posterior pole of the embryonic disc elongates by forming the posterior gastrula extension (PGE, Viebahn et al. 2002). The PGE is characterised by reduced cell density in comparison to the anterior half of the disc, named the anterior gastrula plate (AGP, Plöger and Viebahn 2018) and histologically by lower epiblast cells than in the AGP. At this stage, the top layer, the mural trophoblast, starts to disappear by apoptosis (Rauber 1875; Williams and Biggers 1990). At stage 3, an increasing cell density in the midline of the posterior embryonic disc indicates primitive streak formation (PS, see i.a. Viebahn 1995), histologically defined by the presence of bottle cells in the epiblast and of mesoderm cells in the widening extracellular space between epiblast and hypoblast (Viebahn et al. 1995b).

### Generation of rabbit cDNA and RNA probes

To generate the digoxigenin-labelled mRNA probes of *eomes*, *tbx6* and *pkdcc*, protocols were followed according to the manufacturer’s instructions: The RNA of early rabbit embryos at stage 0–3 (5.2–6.5 dpc) was extracted using RNeasy Mini Kit (Qiagen, Hilden, Germany) adjusted to the weight of the embryo and was then reverse transcribed into cDNA. To amplify the genes of interest, primers were designed using the sequences published by Ensembl and GenBank (*pkdcc*: ENSOCUT00000002998/GenBank XM008254480.1; *eomes*: ENSOCUT00–000000452/GenBank XR518364.1; *tbx6*: ENSOCUT00000026488.1/GenBank XM008257944.1). Successful primer combinations result in a 817 bp fragment of *pkdcc* corresponding to nucleotides 223–1040 (forward primer: 5’- TCGGTCCTCAACGTGCTCTTC -3′ and reverse primer: 5’- GCATTATTGCACGTTTGTCCTGG -3′), in a 949 bp fragment of *eomes* (transcript variant 1) corresponding to nucleotides 31-980 showing 99% identity to all transcript variants (forward primer: 5’- TCGGTCCTCAACGTGCTCTTC -3′ and reverse primer: 5′- GCATTATTGCACGTTTGTCCTGG -3′), and in a 246 bp fragment of *tbx6* (transcript variant 1) corresponding to nucleotides 2393-2147 showing 99% identity with the transcript variant 2. The PCR products were cloned in GEM®-T Easy (Promega, Mannheim, Germany) and sequenced on both strands to confirm the product as correct cloned genes. The sequence for *wnt3* probe had been generated by Idkowiak (2007). mRNA probes were built using plasmids as described by Püschel and Jouneau (2014).

### In situ hybridisation and histology

 Published protocols (Püschel and Jouneau 2014) were used for in situ hybridisation, embedding in Technovit 8100® (Heraeus-Kulzer, Wehrheim, Germany), and sectioning of embedded tissues. Specific mRNA binding was made visible using BM-purple staining solution (Boehringer, Mannheim, Germany) at room temperature in the dark. Depending on  specific and background staining intensities, staining reactions were stopped between the second and the tenth day of incubation and each embryo was spread in Mowiol 4-88 (Hoechst, Frankfurt, Germany) under a cover glass. After a photograph was taken with a stereomicroscope (Zeiss, Göttingen, Germany), embryos were embedded in Technovit^®^ (Heraeus-Kulzer, Wehrheim, Germany) and cut in 5-μm-thin sagittal sections using a Leica 2050 SuperCut microtome. Sections were analysed at high magnification using differential interference contrast (DIC). At least three replicates were obtained per stage and the most representative of the three was chosen to be presented in a Figure.

## Results

In the following, the expression patterns of the four genes analysed are compared in juxtaposition: in a first subsection, at the ‘macroscopical’ level, and in a subsequent subsection, at the histological level and concentrating on the anterior and posterior embryonic disc borders. In this way, subtle differences between expression patterns can be compared easily in particular embryonic disc domains, on the one hand, in cell layers, on the other.

### Gross morphology of expression patterns

**Fig. 1:**
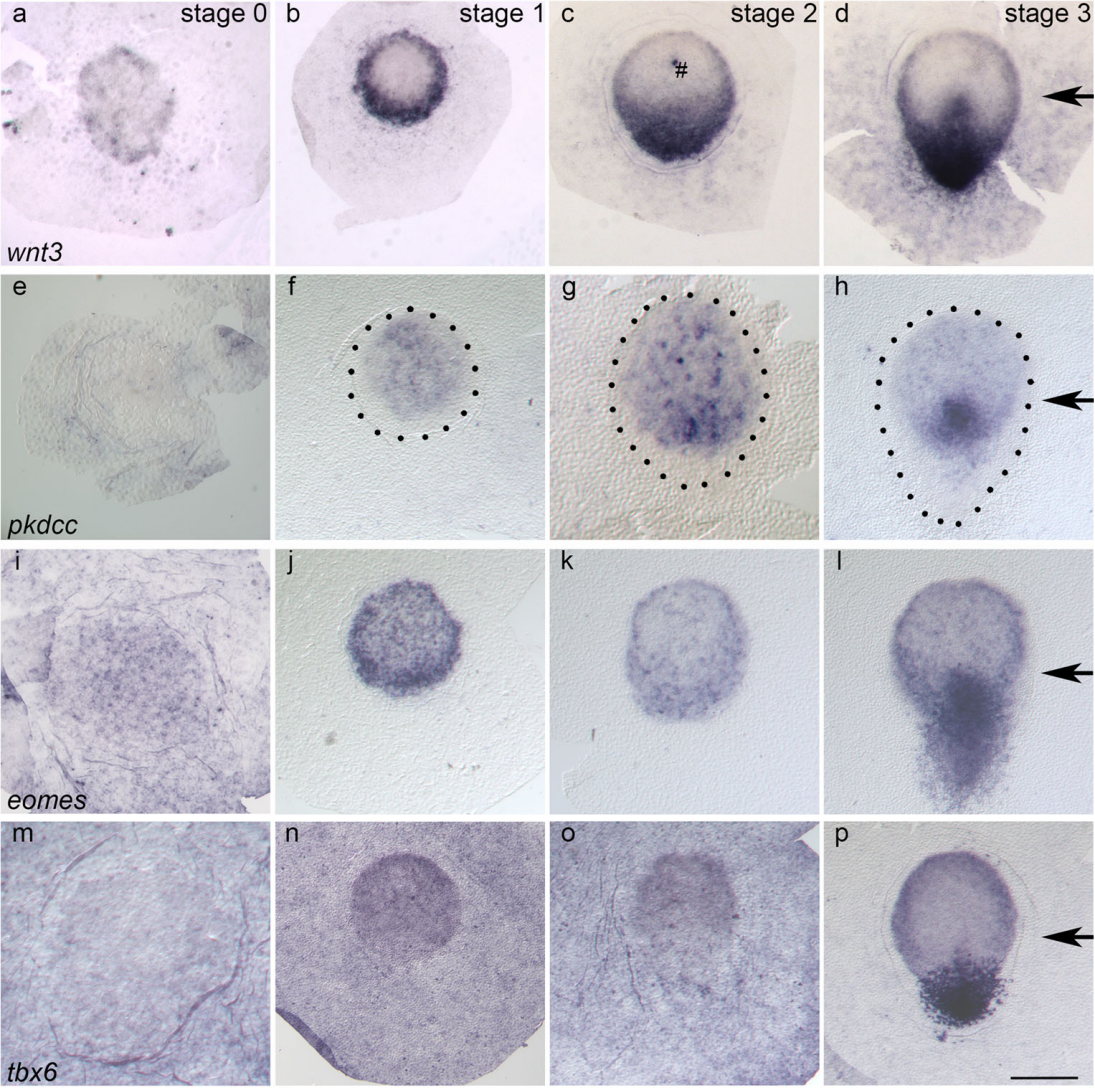
Expression patterns of *wnt3* (**a**–**d**), *pkdcc* (**e**–**h**), *eomes* (**i**–**l**) and *tbx6* (**m**–**p**) in en face views at stages 0 (**a**, **e**, **i**, **m**), 1 (**b**, **f**, **j**, **n**), 2 (**c**, **g**, **k**, **o**) and 3 (**d**, **h**, **l**, **p**) using bright-field illumination. Embryonic discs are oriented with the anterior pole to the top. Stages are indicated by Arabic numbers; genes names are printed in italics. Dotted lines (**f**, **g**, **h**) mark the border of the embryonic disc as determined by cell density in dark-field microscopy (see suppl. Fig. 1). Black arrows mark the tip of the primitive streak. The hash symbol (**c**) indicates a staining artefact. The scale bar corresponds to 25 μm in panels showing stage 0 embryonic discs (**a**, **e**, **i**, **m**), to 450 μm (**g**), 550 μm (**o**), and 500 μm (**b**–**d**, **f**, **h**, **j**–**l**, **n**, **p**) in panels showing  later stages, respectively

At stage 0, single *wnt3* expressing cells are found in the centre of the embryonic disc (n = 9, Fig. 1a), and groups of *wnt3* expressing cells are seen in the periphery of the embryonic disc. These groups form a slightly broader domain at one pole (Fig. 1a, bottom) when compared to the other pole (Fig. 1a, top). The extraembryonic tissue lacks *wnt3* expression completely. At stage 1 (n = 12, Fig. 1b), the peripheral cells of the embryonic disc strongly express *wnt3* revealing a ring-like domain which differs in its width and with its intensity at two poles: One pole, which can be defined as the anterior pole by its cell density and by its sharp margin towards the extraembryonic tissue (Suppl. Fig. 1b), shows a weaker expression and a thinner domain in comparison to the opposite and, thus, posterior pole. Close to the embryonic disc, single cells express *wnt3* in the extraembryonic region. At stage 2 (n = 5, Fig. 1c), the elongated embryonic disc reveals a broad expression domain correlating with the location of the PGE. In this domain, the intensity of expression increases towards the posterior pole. At stage 3 (n = 6, Fig. 1d), single *wnt3* expressing cells in the anterior margin form a dotted domain, which together with the persisting posterior domain is reminiscent of a basket shape. This basket-like domain is supplemented by an expression domain correlating with the anterior part of the primitive streak. As at the previous stages, a few cells in the extraembryonic tissue express *wnt3*.

**Fig. 2:**
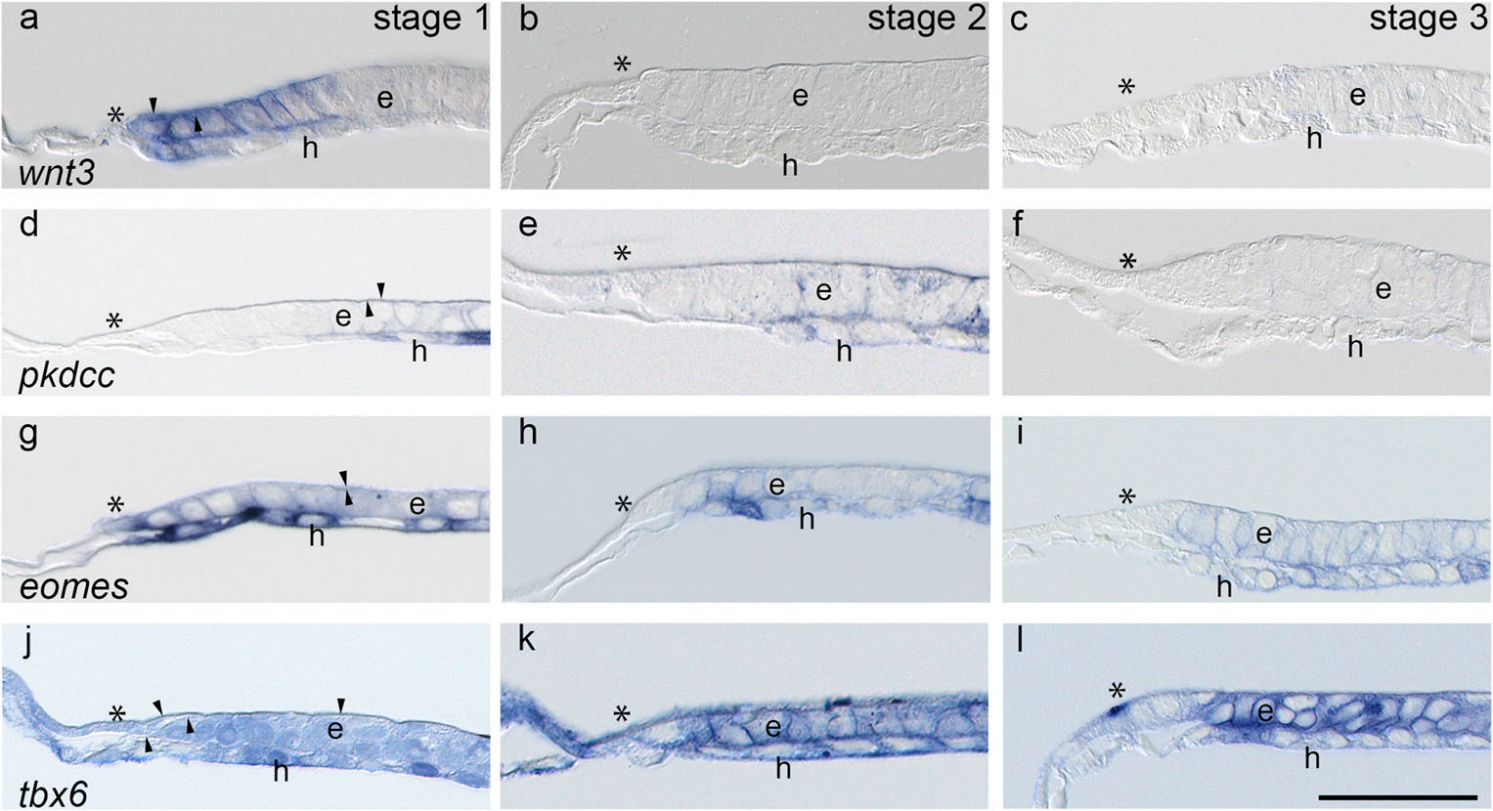
Near-median sagittal Technovit^(R)^ sections of *wnt3* (**a**–**c**), *pkdcc* (**d**–**f**), *eomes* (**g**–**i**), and *tbx6* (**j**–**l**) expression domains in the anterior margin at stages 1 (**a**, **d**, **g**, **j**), 2 (**b**, **e**, **h**, **k**) and 3 (**c**, **f**, **i**, **l**). Anterior is to the left, asterisks indicate the border of the embryonic disc  as determined by the most anterior epiblast cell. Arrow heads point to Rauber cells (**a**, **d, g,** **j**). e: epiblast, h: hypoblast . Scale bar : 200 μm

*Pkdcc* is expressed in the embryonic disc (and not extraembryonically) throughout the stages of interest and complementarily, at first, to *wnt3* (Fig. 1e–h): At stage 0 (n = 3, Fig. 1e), a faint *pkdcc* expression is seen in some specimens, while stage 1 (n = 4, Fig. 1f) reveals an almost homogeneous weak expression pattern in a central circular area slightly shifted anteriorly which contains a few dots representing single cells and excludes the ring-like domain of *wnt3* (cf. Figs. 1b, f and 2a, d described below). At stage 2 (n = 3, Fig. 1g), the dotted expression in the anterior half of the disc increases in both intensity and density, containing a densely dotted, semi-circular domain in the posterior third of the circular area near the border between the AGP and the PGE (as indicated by cell density, see suppl. Fig. 1g). At stage 3 (n= 3, Fig. 1h), *pkdcc* is still weakly expressed in the AGP but shows a strong domain which correlates with the tip of the primitive streak and merges with a weak sickle-like domain at the posterior margin of the AGP.

*Eomes* expression becomes distinct during axis formation and shows similarities to the expression of *wnt3* (Fig. 1i–l): Both the embryonic disc and the extraembryonic tissue show a dotted expression pattern at stage 0 (n = 5, Fig.1i). From stage 1 (n = 3, Fig. 1j) onwards, the expression in the extraembryonic tissue is completely lost, whereas in the embryonic disc, the margin shows a stronger expression than the centre. This uneven ring-like domain is broader at the posterior pole than at the anterior pole (defined by morphological characteristics in sagittal sections, see below) and thus appears to be similar to the expression of *wnt3*. At stage 2 (n = 4, Fig. 1k), the uneven ring-like domain takes on a basket-like shape with a thin ‘handle’ at the anterior margin of the embryonic disc and a broad domain at the PGE; the expression in centre of embryonic disc is of reduced intensity compared to the previous stage. At stage 3 (n = 4, Fig. 1l), the posterior part of the basket-like domain is further transformed into a Y-shape which correlates with the primitive streak and the posterolateral margins of the AGP. The expression in the centre of embryonic disc is still present but still with a lower intensity than at stage 1.

*Tbx6* is expressed at all stages investigated here (Fig. 1m–p) including in the extraembryonic tissues between stages 0 and 2. At stage 0 (n = 3, Fig. 1m), there is a weak uniform expression both embryonically and extraembryonically, and at stages 1 (n = 6, Fig. 1n) and 2 (n = 4, Fig. 1o), there is a gradient in the embryonic disc between a weak expression posteriorly and strong expression anteriorly, and the anterior margin is accentuated by a higher intensity than in the remaining embryonic disc. At stage 2, the weak posterior expression domain is broader than at stage 1 and correlates roughly with the shape of the PGE. At stage 3 (n = 3, Fig. 1p), a strong expression domain, similar to the shape of an inverted triangle, covers the posterior half of the primitive streak and the bilaterally adjacent areas of the embryonic disc and proximal parts of the extraembryonic tissue. At the anterior margin, a sickle-shaped, strongly *tbx6* expressing domain is distinguishable from a weak *tbx6* expressing domain in the centre of the embryonic disc.

### Germ layer expression domains

In the anterior margin (Fig. 2), at stage 1, histologically defined by cuboidal hypoblast cells of the AMC, up to six rows of epiblast cells, a few remaining Rauber’s cells and a couple of hypoblast cells all express *wnt3* strongly (Fig. 2a), whereas *pkdcc* (Fig. 2d) is expressed weakly in the epiblast and strongly in the hypoblast posteriorly adjacent to the *wnt3* expression domain. *Eomes* and *tbx6*, too, are expressed in all layers of the anterior margin at stage 1, whereby *eomes* (Fig. 2g) shows a broader and stronger expression domain in the hypoblast than in the epiblast cells, and *tbx6* (Fig. 2j) is evenly distributed within epiblast, hypoblast and adjacent extraembryonic tissues. At stage 2, the cells of the AMC and the overlying epiblast cells lack *wnt3* expression completely (Fig. 2b), while *pkdcc* is still weakly expressed in both epiblast and hypoblast as before (Fig. 2e). The thin anterior expression domain of *eomes* seen in dorsal views of the entire embryonic disc (Fig. 1k) correlates histologically with *eomes* expression in first three rows of anterior hypoblast cells (Fig. 2h). In the remaining hypoblast, single cell expression matches the dotted expression pattern seen centrally in the dorsal views (Fig. 1j and k). *Tbx6* (Fig. 2k) is uniformly expressed in all layers whereby the expression in the trophoblast and the yolk sac epithelium confirms (as at stage 1) the homogeneous extraembryonic expression seen in the dorsal views (Fig. 1m–o). At stage 3, epiblast and hypoblast cells near the AMC express *wnt3* weakly or not at all (Fig. 2c) and lack an expression of *pkdcc* completely (Fig. 2f). *Eomes* is evenly expressed in both anterior epiblast and hypoblast sparing, however, the most anterior cells in the transitional zone between extraembryonic and embryonic tissues (Fig. 2i). *Tbx6*, in contrast, shows a strong expression in both anterior epiblast and hypoblast and a weak expression in the epiblast cells of transitional zone (Fig. 2l). The extraembryonic tissue lacks *tbx6* expression at this stage (cf. dorsal view, Fig. 1p).

**Fig. 3:**
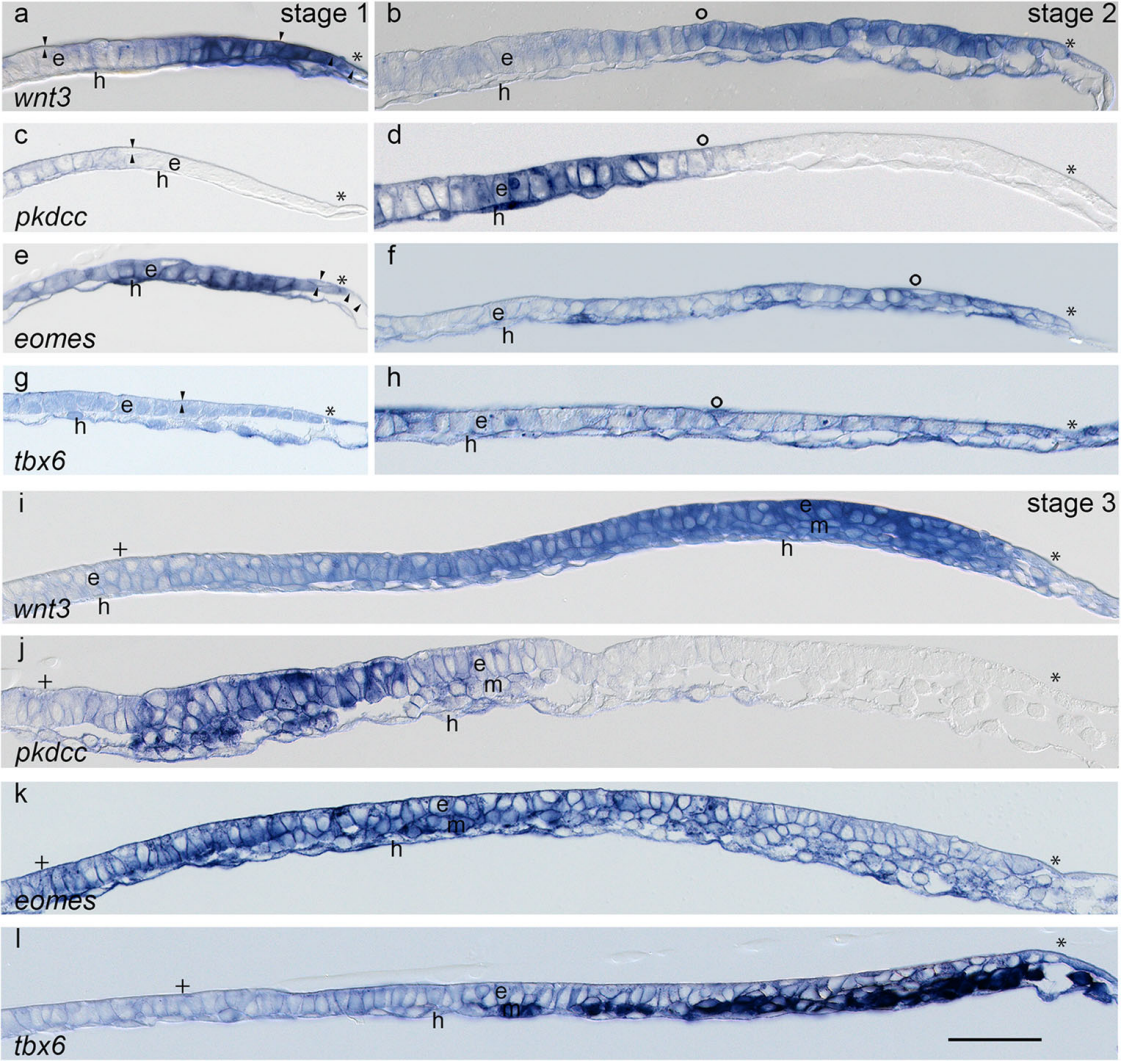
Near-median sagittal Technovit^(R)^ sections of *wnt3* (**a, b, i**), *pkdcc* (**c, d, j**), *eomes* (**e, f, k**) and *tbx6* (**g**, h, **l**) expression domains in the posterior margin at stages 1 (**a**, **c**, **e**, **g**), 2 (**b**, **d**, **f**, **h**) and 3 (**i**, **j**, **k**, **l**). Orientation of sections and inscriptions are as in Fig. 2; asterisks, however, indicate the posterior border of the embryonic disc. The ring symbol indicates the border between the anterior gastrula plate (AGP) and the posterior gastrula extension (PGE). The plus symbol marks bottle cells.  m: mesoderm cells . Scale bar (**l**): 200 μm

While the posterior margin (Fig. 3) elongates during axis formation, its changing composition of germ layers expresses the genes of interest in the following way: At stage 1, *wnt3* is expressed strongly in all three layers of the posterior margin (Rauber’s layer, epiblast and hypoblast, Fig. 3a). Anterior to this expression domain and coinciding with the emergence of the columnar cells in the epiblast, the hypoblast lacks *wnt3* expression, whereas the columnar epiblast cells express *wnt3* weakly. *Pkdcc* (Fig. 3c) is weakly expressed in all three layers anterior to the *wnt3* expression domain. In comparison to its expression in the anterior margin (Fig. 2d–f), *pkdcc* expression shows a higher intensity in the epiblast and a weaker intensity than in the hypoblast. *Eomes* expression is particularly strong in both epiblast and hypoblast in a domain similar to the strong *wnt3* expression domain; it continues with a weaker intensity towards the anterior pole (Fig. 3e). *Tbx6* at stage 1 (Fig. 3g) is weakly expressed in all layers of the whole posterior margin and in the adjacent extraembryonic tissue. At stage 2, *wnt3* (Fig. 3b) is expressed stronger in the cuboidal epiblast cells and underlying hypoblast cells of the PGE than in the columnar epiblast cells and underlying hypoblast cells of the AGP. In contrast, *pkdcc* (Fig. 3d) shows a strong expression in a few of the most posterior columnar epiblast cells and underlying hypoblast cells of the AGP and lacks expression in the PGE (where the strong *wnt3* expression had appeared). *Eomes* (Fig. 3f) is expressed stronger in the germ layers of the PGE than in the layers of the AGP, and *tbx6* (Fig. 3h) shows a uniform expression pattern in all layers on both sides of the posterior margin. At stage 3, *wnt3* expression (Fig. 3i) is weak in all germ layers of the region where the most anterior bottle cells and mesoderm cells appear, but it is strong in all layers of the region where mesoderm cells have formed a multi-layered structure of the embryonic disc by this stage. Close to the posterior border of the embryonic disc, however, *wnt3* expression is only weak. Again in contrast to *wnt3*, *pkdcc* expression marks both epiblast and mesoderm strongly in the region of the most anterior mesoderm cells (Fig. 3j), and this domain is surrounded by weakly *pkdcc*-expressing cells including epiblast, bottle cells, mesoderm and hypoblast cells. Similar to *pkdcc* expression, the region of the most anterior mesoderm cells shows a particularly strong expression of *eomes* in epiblast, mesoderm and hypoblast cells (Fig. 3k). The intensity of the *eomes* expression decreases in an anterior to posterior fashion, while there is no expression beyond the posterior pole of the embryonic disc. *Tbx6* expression (Fig. 3l) is confined to the posterior third of the primitive streak (cf. Fig. 1p) and, in contrast to *eomes*, increases in intensity in the epiblast and hypoblast towards the posterior pole; here, mesoderm cells express *tbx6* with a particularly strong intensity. The sections reveal also that the dotted expression pattern in the extraembryonic tissue seen in the dorsal view of the whole embryonic disc (Fig. 1p) correlates with emigrated mesoderm cells strongly expressing *tbx6* and lying between trophoblast and yolk sac epithelium.

## Discussion

**Fig. 4:**
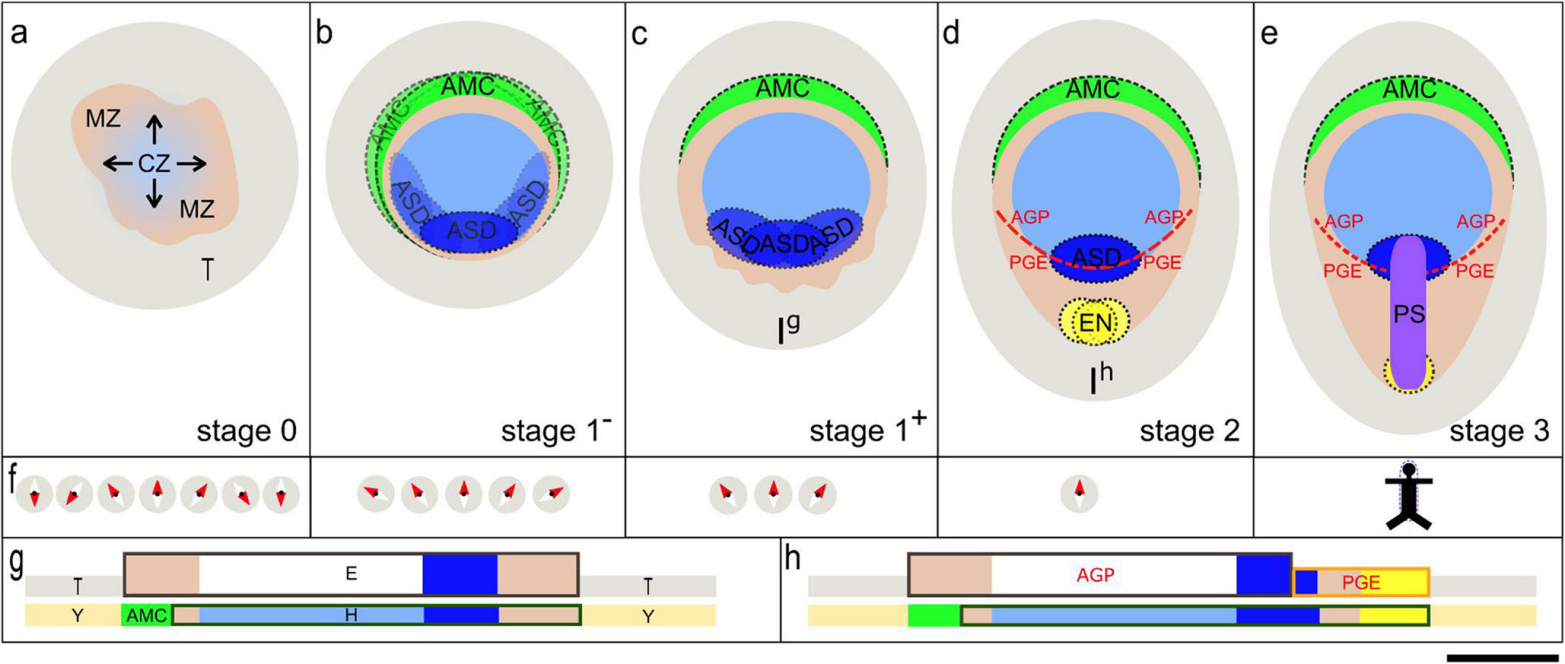
Schematic representation of mammalian initial a.-p. axis formation stages in dorsal views (**a**–**e**) and median sagittal sections (**g** and **h**) using the rabbit as a model and compass needle symbols (**f**) to illustrate the increasing restriction of possible a.-p. axis orientation in the TAP model, in which  AMC, ASD and EN are the  putative anchors 1, 2, and 3, respectively. T: trophoblast (grey), Y: yolk sac epithelium (orche), E: epiblast (red, boxed in upper layer), H: hypoblast (boxed in lower layer), MZ: marginal zone (camel), CZ: central zone (light blue), AMC: anterior marginal crescent (light green), ASD: anterior streak domain (dark blue), red dashed line: border between anterior gastrula plate (AGP) and posterior gastrula extension (PGE), EN: end node (yellow), PS: primitive streak (purple), arrows: expansion of CZ, stick figure superimposed on PS in **f**, right hand panel: contribution of PS to adult body parts. Scale bar: roughly 500 μm

The four genes selected for this study reveal specific axis and cell layer-related expression patterns and may be relevant for the models considered here in the following way: *Wnt3* and *eomes* are expressed in both epi- and hypoblast in a marginal zone (MZ, Fig. 4) at the beginning of axis formation (stage 1), and at mid-axis formation (stage 2), they show a basket-like pattern complementary to *pkdcc* expression in the hypoblast of a central zone (CZ, Fig. 4). Posteriorly, on the border between AGP and PGE at stages 2 and 3, *pkdcc* is also expressed in the epiblast and marks the (forming) ASD. Prior to axial differentiation, *tbx6* expression (in both layers) shares the almost uniform expression pattern with *eomes* and accentuates its bias towards the anterior pole until an anterior sickle-shaped domain is definable distinct from the remainder of the embryonic disc. The PS (at stage 3) is marked (1) by *pkdcc* anteriorly and in all layers, (2) by *tbx6* posteriorly in mesoderm cells and (3) by different intensities of *wnt3* and *eomes* expression in its entirety and in all layers.

Comparing these expression patterns with previous results obtained in mammalian embryos, namely rabbit and mouse, there seem to be mostly gradual differences. W*nt3* expression described to start at stage 2 and not a stage 0 in the rabbit by Yoshida et al. (2016) is in contrast to the present study and may be explained by (1) the dedicated low-background protocol of the present study, which allows exposure to the staining reaction for more than a week, and (2) by the direct comparison of sagittal sections at early stages which reveal subtle but stage-specific histological characteristics of the anterior margin (cf. Fig. 2a and b). Histological analysis of the specimen shown in Fig. 7Ai of Yoshida et al. (2016) may, in fact, show this specimen to be a stage 1 embryo. This would also be in line with the appearance of *wnt3* expression in the mouse at a corresponding developmental stage (E5.57, Fig. 5c in Rivera-Perez and Magnuson 2005). In contrast to the diffuse and almost circular *wnt3* expression pattern in the rabbit during early axis formation (stages 0 and 1), *wnt3* appears in the mouse at the future posterior pole and spreads anteriorly proximal to the AVE (Rivera-Perez and Magnuson 2005), but in the absence (so far) of evidence in earlier stages of the rabbit showing similar dynamics, the two patterns may be considered principally circular especially when the egg-cylinder is schematically flattened (cf. Behringer et al. 2000). In addition, *pkdcc* appearing in the epiblast and coinciding with the centre of the ‘mature’ *nodal* domain (i.e. the ASD) is in line with a similarly positioned *pkdcc* expression domain in the mouse (Imuta et al. 2009) and, therefore, seems to be evolutionarily conserved, too. Functional implications of this close association between *wnt* and *pkdcc* may become apparent with further interspecific comparisons, namely with chick (Skromne and Stern 2001), mouse (Perea-Gomez et al. 2004; and Rivera-Perez and Magnuson 2005) and Xenopus (Ding et al. 2017) in that *wnt* domains are involved in inducing the organiser marked by *pkdcc* (Ding et al. 2017) during gastrulation. Circular *eomes* expression in the rabbit fits the circular (extraembryonic) ectoderm domain of *eomes* (Ciruna and Rossant 1999; Russ et al. 2000) and of the further T-box gene *brachyury* (Perea-Gomez et al. 2004; Rivera-Perez and Magnuson 2005*)*, and it fits the circular expression domain in the extraembryonic border of the area pecullida in the chick (Pernaute et al. 2010). It also corresponds to a region lying peripherally to *wnt* in both chick (Hume and Dodd 1993; Lee et al. 2020) and mouse (Liu et al. 1999; Rivera-Perez and Magnuson 2005), whereas in the rabbit, the overlapping ring-like expression of *eomes* and *wnt3* is restricted to the embryonic disc. However, the ring-like patterns of *wnt* and *eomes* in the hypoblast may also be taken as signs of an extraembryonic fate of these cells in the margin of the embryonic disc in the rabbit (cf. Blomberg et al. 2008) and of the ‘soft transition’ between extra- and embryonic tissue in the lower layer of the embryo. Posterior *tbx6* expression in the rabbit, finally, is shared with the patterns observed in the chick (Lee et al. 2020; Torlopp et al. 2014) and mouse (Chapman et al. 1996), and the lack of anterior *tbx6* expression in the chick may mirror the principle of inverted axis formation when comparing rabbit and chick with regard to *pitx2*, too (Plöger and Viebahn 2018). The lack of anterior *tbx6* expression at the early streak stage in the mouse (Chapman et al. 1996), however, may be due to the specific requirements of the egg-cylinder shape where the posterior pole (containing the PS) has an intriguingly close topographical relationship with the anterior pole (containing the AVE).

Regarding the TAP model in the rabbit embryo, the almost simultaneous development of the first anchor, the AMC, and of the second anchor, the ASD, (Fig. 4b and c) as indicated by the early *pkdcc* expression in the epiblast is well suited to narrow down the number of possible orientation angles of the a.-p. axis at stage 2 (Fig. 4f) and may thus define the direction of a.-p. elongation, a process also observed in connection with the dynamics of *brachyury* expression in the mouse (Rivera-Perez and Magnuson 2005). Functionally, *pkdcc* may play a role in cell movement regulation in the region of marked embryonic disc elongation (cf. Fig. 1k) through its involvement in the planar cell polarity pathway (Vitorino et al. 2015). The third anchor point, marked by *tbx6* expression (Fig. 4d) and coinciding with *bmp4* and *blimp1* expression (Hopf et al. 2011), is reminiscent of the dense accumulation of mesoderm cells in the posterior part of the PS termed ‘end node’ in the human embryo (Florian 1933). Shortly before PS formation, the appearance of the third anchor (Fig. 4d) together with the ASD may thus fix the angle of the PS (Fig. 4e). A putative concentric system prior to the TAP model may consist of a marginal zone (MZ, Fig. 4a) marked by the ring-like patterns of *wnt3*, *eomes*, *pitx2* (Plöger and Viebahn 2018), *bmp2* and *bmp4* (Hopf et al. 2011) and of a central zone (CZ, Fig. 4a) marked by the early *pkdcc* expression in the hypoblast centrally and partially by the expression of further inhibitors such as *dkk* and *cerl* (Idkowiak et al. 2004). Thereby, the genes expressed in the MZ may be involved in organiser induction and mesoderm formation (Schier 2003; Houston and Wylie 2004) indicating the potential of the margin for PS formation with a symmetric marginal zone allowing multiple a.-p. axes to arise (represented by the compass needles in Fig.4f). The first molecular a.-p. polarisation in the rabbit appearing through different domain widths in the marginal zone (Abb. 1a–c, or 1j–k, modelled in Fig. 4a–c) could then be caused by (1) the interaction between these zones, (2) different growth rates within the entire embryonic disc or (3) gradients such as shown by *tbx6* expression. Taken together, the data presented here may provide new ideas for early axis formation and, supporting the TAP model for later steps of axis formation, may lead to the search for these anchors in other amniote model organisms, especially in those with a flat embryonic disc.

## Supplementary information

Suppl. Fig. 1 Expression pattern of *wnt3* (a-d), *pkdcc* (e-h), *eomes* (i-l) and *tbx6* (m-p) in en face views of the same specimens shown in Fig. 1 at stage 0 (a,e,i,m), 1 (b,f,j,n), 2 (c,g,k,o) and 3 (d,h,l,p) using dark-field illumination. Orientation,  inscriptions and the scale bar are as in Fig. 1. Note the edges of the extraembryonic tissue standing out against the black background in some of the smaller specimens.(PNG 3064 kb)

High Resolution (TIF 8072 kb)

## Data Availability

Available
