# Supplementary figures and images for: Expression patterns of signalling molecules and transcription factors in the early rabbit embryo and their significance for modelling amniote axis formation
Source: Dev Genes Evol. 2021 Jun 7;231(3-4):73–83. doi: 10.1007/s00427-021-00677-w (PMC8213660; doi:10.1007/s00427-021-00677-w)

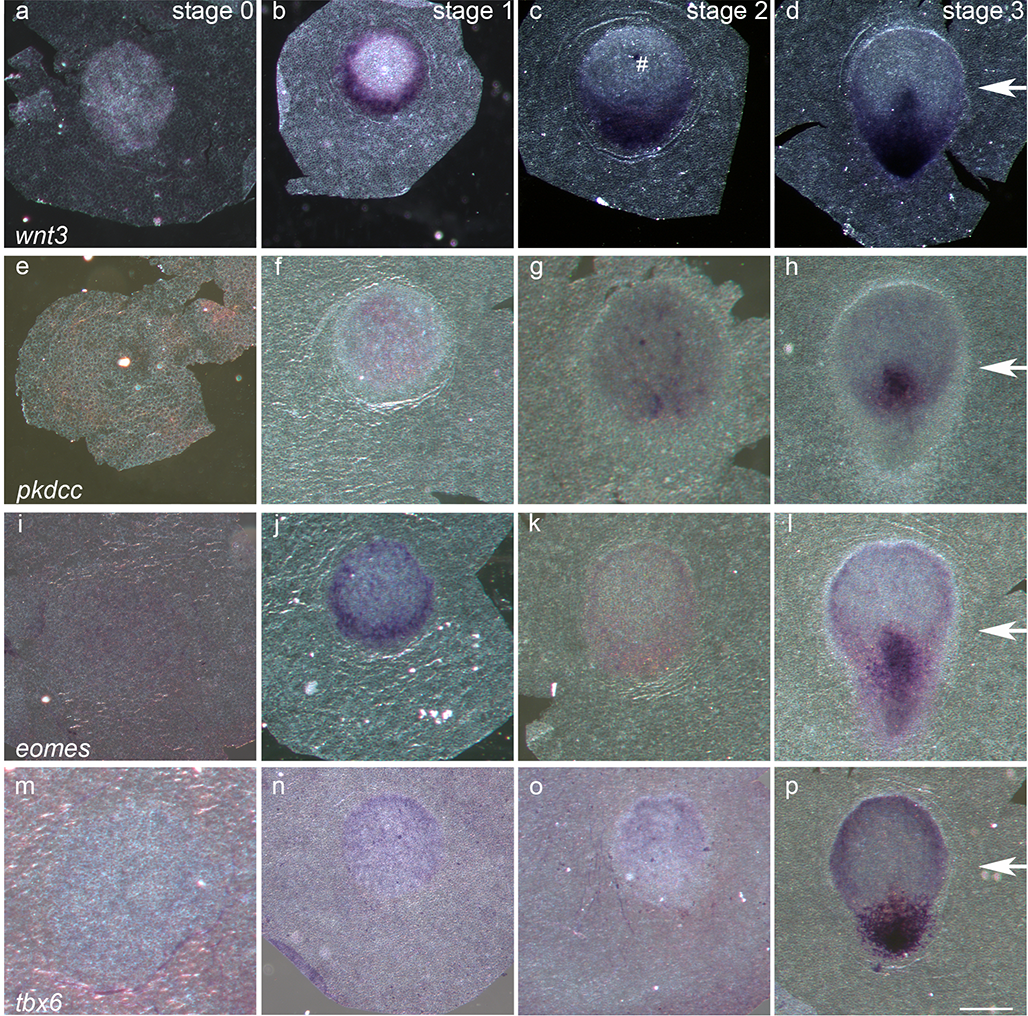

Supplement: Supplementary file 1 — Expression pattern of wnt3 (a-d), pkdcc (e-h), eomes (i-l) and tbx6 (m-p) in en face views of the same specimens shown in Fig. 1 at stage 0 (a,e,i,m), 1 (b,f,j,n), 2 (c,g,k,o) and 3 (d,h,l,p) using dark-field illumination. Orientation, inscriptions and the scale bar are as in Fig. 1. Note the edges of the extraembryonic tissue standing out against the black background in some of the smaller specimens.(PNG 3064 kb) [file 427_2021_677_Fig5_ESM.png]

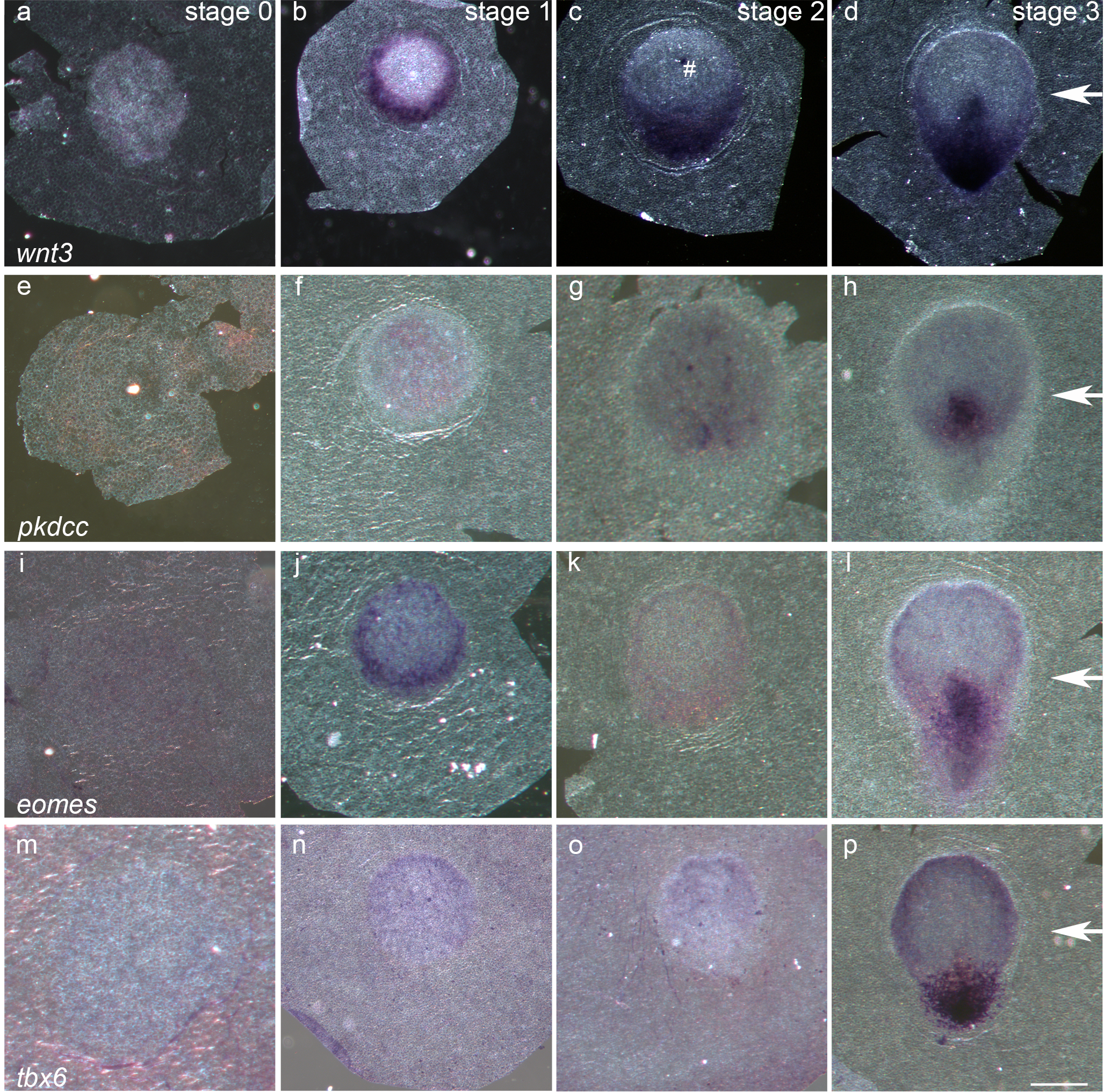

Supplement: Supplementary file 2 — High Resolution (TIF 8072 kb) [file 427_2021_677_MOESM1_ESM.tif]
